# Supplementary material for: Functional connectivity in infants’ visual cortex and its links to motion processing and autism
Source: Sci Rep. 2026 Feb 28;16:7826. doi: 10.1038/s41598-026-42048-3 (PMC12953626; doi:10.1038/s41598-026-42048-3)
Supplement: Supplementary file 3 — Supplementary Material 3 [file 41598_2026_42048_MOESM3_ESM.docx]

**SUPPLEMENTARY INFORMATION**

**Supplementary Table 1:** Analysis-level exclusions for functional connectivity calculation

**Supplementary Table 2:** Analysis-level exclusions for global motion laterality analysis

**Supplementary Table 3:** Descriptives of infants in functional connectivity dataset

**Supplementary Table 4:** Descriptives of infants in global motion laterality dataset

**Supplementary Table 5:** Results of statistical tests and models presented in the main text and Supplementary Information

**Supplementary Figure 1:** Group analysis of dbWPLI Near & Far Connectivity variables in six experimental conditions

**Supplementary Figure 2:** Permutation tests of across-group differences in five experimental conditions based on the flipped topographies

**Supplementary Figure 3:** Across-stimulus topographic connectivity differences in each frequency band and group

**Supplementary Video 1:** 1-minute video clip used for social stimulus (separately attached video file)

**Supplementary Video 2:** 1-minute video clip used for non-social stimulus (separately attached video file)

**Supplementary Table 1 | Analysis-level exclusions for functional connectivity calculation**

|  | **Sample size** |
| --- | --- |
| Initial sample | 119^†^ |
| No video coding, no EEG data | -2 |
| Sample having EEG & video coding | 117 |
| Bad EEG data & rejected by HAPPE for preprocessing | -3 |
| Sample pre-processed by HAPPE | 114 |
| Exclusion due to no looking to stimuli | -12 |
| Sample going into analysis stage | 102 |
| Exclusion due to the 90-epoch threshold | -34 |
| **Final sample for computing dbWPLI variables** | **69** |
| Sub-sample with ADOS-2 CSS at 36 mo. | 59 |
| Sub-sample with Global Motion Laterality Score & ADOS-2 CSS at 36 mo.^‡^ | 43 |

^†^The same participants as in ref.^1^ plus 27 new infants, after excluding ADHD-only cases

^‡^Crf. Supplementary Table 2

**Supplementary Table 2 | Analysis-level exclusions for global motion laterality analysis^1^**

|  | **Sample size** |
| --- | --- |
| Initial sample | 119^†^ |
| No EEG data | -1 |
| Sample having EEG | 118 |
| Bad EEG data | -23 |
| **Final sample for computing Global Motion Laterality Score** | **95** |
| Sub-sample with ADOS-2 CSS at 36 mo. | 82 |
| Sub-sample with Global Motion Laterality Score & ADOS-2 CSS at 36 mo.^‡^ | 43 |

^†^The same participants as in ref.^1^ plus 22 additional infants

^‡^Crf. Supplementary Table 1

**Supplementary Table 3 | Descriptives of infants in functional connectivity dataset**

| ***EEG recording at 5 months*** | **EL** | | **LL** |
| --- | --- | --- | --- |
| N | 49 | | 20 |
| Female | 31 | | 9 |
| Age in months (mean + s.d.) | 5.51 + 0.66 | | 5.33 + 0.44 |
| MSEL^*^ Early Learning Composite Score | 96.9 + 11.1 | | 97.9 + 10.0 |
| ***ADOS-2 at 36 months*** | **EL High-ADOS** | **EL Low-ADOS** | **LL** |
| N | 11 | 28 | 20 |
| Female | 4 | 17 | 11 |
| Age in months (mean + s.d.) | 37.80 + 1.95 | 37.85 + 2.75 | 37.65 + 3.02 |

*Mullen Scales of Early Learning (MSEL)^2^

**Supplementary Table 4 | Descriptives of infants in global motion laterality dataset^1^**

| ***EEG recording at 5 months*** | **EL** | | **LL** |
| --- | --- | --- | --- |
| N | 71 | | 24 |
| Female | 35 | | 15 |
| Age in months (mean + s.d.) | 5.44 + 0.72 | | 5.39 + 0.62 |
| MSEL^*^ Early Learning Composite Score | 96.3 + 10.3 | | 102.1 + 7.4 |
| ***ADOS-2 at 36 months*** | **EL High-ADOS** | **EL Low-ADOS** | **LL** |
| N | 19 | 44 | 19 |
| Female | 6 | 26 | 12 |
| Age in months (mean + s.d.) | 38.10 + 2.65 | 37.98 + 2.84 | 37.46 + 2.84 |

*Mullen Scales of Early Learning (MSEL)^2^

**Supplementary Table 5 | Results of statistical tests and models presented in the main text, Extended Data, and in Supplementary Information**

All analyses presented in the manuscript were performed using standard functions/scripts in R language. All *p*-values are two-tailed.

***Abbreviations:*** FC_ = Far-Connectivity; NC_ = Near-Connectivity; [Band]NS = NonSocial stimuli; [Band]S = Social stimuli; dbWPLI = debiased Weighted Phase Lag Index; GMLS = Global Motion Laterality Score; EL = Elevated Likelihood for ASD; LL = Low Likelihood for ASD; LR = Likelihood Ratio; npar = no. of model parameters; LogLik = Log-Likelihood; df = degrees of freedom; GFLS = Global Form Laterality Score; LMLS = Local Motion Laterality Score; LFLS = Local Form Laterality Score

| Category | Section in Main Text | Referred to in  Display Item or on Manus. Page | Test / Model | Parameter estimates | *N* | *p* |
| --- | --- | --- | --- | --- | --- | --- |
| Main result | **Results:** *Association between infants’ visual cortical functional connectivity and autistic symptoms in toddlerhood* | Fig. 3a | Bivariate correlation (Pearson’s *r*) | All: *r*(FC_ThetaNS x ADOS2) = .50  EL: *r*(FC_ThetaNS x ADOS2) = .64  LL: *r*(FC_ThetaNS x ADOS2) = -.38 | 59  39  20 | < .001  < .001  .095 |
| Main result | **Results:** *Association between infants’ visual cortical functional connectivity and autistic symptoms in toddlerhood* | Fig. 3a | Main-effect model of  ADOS2 ~ dbWPLI  (OLS regression) | **Regression coefficient [Effect size]**  *β*(sex_Male) = .87 [part-𝜂^2^ = .077]  *β*(age) = -.02 [part-𝜂^2^ = .050]  *β*(status_LL) = -.98 [part-𝜂^2^ = .087]  *β*(F_ThetaNS) = 1.12 [part-𝜂^2^ = .229]  *β*(F_GammaNS) = -.59 [part-𝜂^2^ = .076]  *β*(F_GammaS) = .60 [part-𝜂^2^ = .085]  *β*(N_ThetaNS) = .32 [part-𝜂^2^ = .028]  *β*(N_AlphaS) = .36 [part-𝜂^2^ = .047]  *R*^2^ = .440 | 59 | .047  .111  .034  < .001  .047  .036  .234  .124 |
| Main result | **Results:** *Association between infants’ visual cortical functional connectivity and autistic symptoms in toddlerhood* | Fig. 3a | Interaction-effect model of  ADOS2 ~ dbWPLI*status  (OLS regression) | **Regression coefficient [Effect size]**  *β*(sex_Male) = .71 [part-𝜂^2^ = .054]  *β*(age) = -.02 [part-𝜂^2^ = .036]  *β*(status_LL) = -.98 [part-𝜂^2^ = .085]  *β*(F_ThetaNS) = 1.18 [part-𝜂^2^ = .233]  *β*(F_GammaNS) = -.52 [part-𝜂^2^ = .058]  *β*(F_GammaS) = .45 [part-𝜂^2^ = .043]  *β*(F_ThetaNS*status_LL) = -1.91 [part-𝜂^2^ = .135]  *β*(F_GammaNS*status_LL) = .41 [part-𝜂^2^ = .005]  *β*(F_GammaS*status_LL) = -.16 [part-𝜂^2^ = .001]  *R*^2^ = .493 | 59 | .100  .181  .029  < .001  .079  .158  .008  .634  .823 |
| Main result | **Results:** *Association between infants’ visual cortical functional connectivity and autistic symptoms in toddlerhood* | Fig. 3b,  Ext. Data Fig. 1 | Across-group difference test of dbWPLI variables  (Wilcoxon test + FDR correction)  *Notes:*  ^i)^EL-High ADOS  ^ii)^EL-Low ADOS  ^iii)^LL | **FC_ThetaNS, effect size (Cohen’s *d*)**  EL Hi-ADOS – EL Lo-ADOS: *d* = 1.61  EL Hi-ADOS – LL: *d* = 1.57  EL Lo-ADOS – LL: *d* = -.14  **FC_ThetaS, effect size (Cohen’s *d*)**  EL Hi-ADOS – EL Lo-ADOS: *d* = -.09  EL Hi-ADOS – LL: *d* = .44  EL Lo-ADOS – LL: *d* = .46  **FC_GammaNS, effect size (Cohen’s *d*)**  EL Hi-ADOS – EL Lo-ADOS: *d* = .42  EL Hi-ADOS – LL: *d* = .55  EL Lo-ADOS – LL: *d* = .12  **FC_GammaS, effect size (Cohen’s *d*)**  EL Hi-ADOS – EL Lo-ADOS: *d* = .73  EL Hi-ADOS – LL: *d* = .66  EL Lo-ADOS – LL: *d* = .02  **FC_AlphaNS, effect size (Cohen’s *d*)**  EL Hi-ADOS – EL Lo-ADOS: *d* = .43  EL Hi-ADOS – LL: *d* = 1.06  EL Lo-ADOS – LL: *d* = .53  **FC_AlphaS, effect size (Cohen’s *d*)**  EL Hi-ADOS – EL Lo-ADOS: *d* = .19  EL Hi-ADOS – LL: *d* = .20  EL Lo-ADOS – LL: *d* = .03  **NC_ThetaNS, effect size (Cohen’s *d*)**  EL Hi-ADOS – EL Lo-ADOS: *d* = -.98  EL Hi-ADOS – LL: *d* = -.91  EL Lo-ADOS – LL: *d* = -.06  **NC_ThetaS, effect size (Cohen’s *d*)**  EL Hi-ADOS – EL Lo-ADOS: *d* = -.15  EL Hi-ADOS – LL: *d* = -.17  EL Lo-ADOS – LL: *d* = .02  **NC_GammaNS, effect size (Cohen’s *d*)**  EL Hi-ADOS – EL Lo-ADOS: *d* = -.34  EL Hi-ADOS – LL: *d* = -.01  EL Lo-ADOS – LL: *d* = .36  **NC_GammaS, effect size (Cohen’s *d*)**  EL Hi-ADOS – EL Lo-ADOS: *d* = -.65  EL Hi-ADOS – LL: *d* = -.66  EL Lo-ADOS – LL: *d* = .07  **NC_AlphaNS, effect size (Cohen’s *d*)**  EL Hi-ADOS – EL Lo-ADOS: *d* = -.52  EL Hi-ADOS – LL: *d* = -.37  EL Lo-ADOS – LL: *d* = .25  **NC_AlphaS, effect size (Cohen’s *d*)**  EL Hi-ADOS – EL Lo-ADOS: *d* = .03  EL Hi-ADOS – LL: *d* = -.12  EL Lo-ADOS – LL: *d* = -.16 | 59  11^i)^  29^ii)^  20^iii)^ | 004  .008  .750  .939  .522  .446  .545  .615  .860  .508  .508  .860  .761  .527  .761  .893  .761  .893  .022  .076  .835  .918  .833  .917  .860  .833  .508  .508  .508  .860  .761  .893  .893  .893  .893  .893 |
| Main result | **Results:** *Association between infants’ visual cortical functional connectivity and autistic symptoms in toddlerhood* | Figs. 3c-d  Ext. Data Fig. 2 | Across-group difference test of flipped, norm. AoI connectivity  (Permutation test + FDR correct.)  *Notes:*  ^i)^EL-High ADOS  ^ii)^EL-Low ADOS  ^iii)^LL | **ThetaNS, mean difference [effect size]**  ** EL Hi-ADOS – EL Lo-ADOS*  Step-3L: *M* = -.020 [*d* = -.81]  Step-2L: *M* = -.056 [*d* = -2.36]  Step-1L: *M* = -.018 [*d* = -.74]  Step-1R: *M* = -.094 [*d* = -2.41]  Step-2R: *M* = -.046 [*d* = -1.52]  Step-3R: *M* = .234 [*d* = 4.16]  ** EL Hi-ADOS – LL*  Step-3L: *M* = -.014 [*d* = -.55]  Step-2L: *M* = -.060 [*d* = -2.44]  Step-1L: *M* = -.018 [*d* = -.68]  Step-1R: *M* = -.100 [*d* = -2.44]  Step-2R: *M* = -.025 [*d* = -.78]  Step-3R: *M* = .217 [*d* = 3.67]  ** EL Lo-ADOS – LL*  Step-3L: *M* = .006 [*d* = .29]  Step-2L: *M* = -.004 [*d* = -.22]  Step-1L: *M* = .000 [*d* = .02]  Step-1R: *M* = -.007 [*d* = -.22]  Step-2R: *M* = .021 [*d* = .86]  Step-3R: *M* = -.017 [*d* = -.36]  **ThetaS, mean difference [effect size]**  ** EL Hi-ADOS – EL Lo-ADOS*  Step-3L: *M* = .004 [*d* = .17]  Step-2L: *M* = -.002 [*d* = -.09]  Step-1L: *M* = -.002 [*d* = -.08]  Step-1R: *M* = -.015 [*d* = -.45]  Step-2R: *M* = .026 [*d* = .93]  Step-3R: *M* = -.012 [*d* = -.26]  ** EL Hi-ADOS – LL*  Step-3L: *M* = -.029 [*d* = -1.09]  Step-2L: *M* = -.021 [*d* = -.81]  Step-1L: *M* = .011 [*d* = .43]  Step-1R: *M* = -.014 [*d* = -.38]  Step-2R: *M* = .005 [*d* = .16]  Step-3R: *M* = .048 [*d* = 1.01]  ** EL Lo-ADOS – LL*  Step-3L: *M* = -.033 [*d* = -1.60]  Step-2L: *M* = -.018 [*d* = -.91]  Step-1L: *M* = .013 [*d* = .64]  Step-1R: *M* = .002 [*d* = .06]  Step-2R: *M* = -.022 [*d* = -.92]  Step-3R: *M* = .059 [*d* = 1.64]  **GammaNS, mean difference [effect size]**  ** EL Hi-ADOS – EL Lo-ADOS*  Step-3L: *M* = -.025 [*d* = -1.19]  Step-2L: *M* = -.001 [*d* = -.07]  Step-1L: *M* = -.002 [*d* = -.09]  Step-1R: *M* = -.026 [*d* = -1.04]  Step-2R: *M* = .007 [*d* = .22]  Step-3R: *M* = .048 [*d* = 1.25]  ** EL Hi-ADOS – LL*  Step-3L: *M* = -.028 [*d* = -1.26]  Step-2L: *M* = -.011 [*d* = -.53]  Step-1L: *M* = -.003 [*d* = -.16]  Step-1R: *M* = -.001 [*d* = -.03]  Step-2R: *M* = -.018 [*d* = -.56]  Step-3R: *M* = .060 [*d* = 1.48]  ** EL Lo-ADOS – LL*  Step-3L: *M* = -.003 [*d* = -.16]  Step-2L: *M* = -.009 [*d* = -.60]  Step-1L: *M* = -.001 [*d* = -.10]  Step-1R: *M* = .026 [*d* = 1.23]  Step-2R: *M* = -.024 [*d* = -.98]  Step-3R: *M* = .012 [*d* = .39]  **GammaS, mean difference [effect size]**  ** EL Hi-ADOS – EL Lo-ADOS*  Step-3L: *M* = -.025 [*d* = -1.21]  Step-2L: *M* = -.013 [*d* = -.76]  Step-1L: *M* = -.009 [*d* = -.52]  Step-1R: *M* = -.044 [*d* = -1.83]  Step-2R: *M* = .024 [*d* = .90]  Step-3R: *M* = .067 [*d* = 1.97]  ** EL Hi-ADOS – LL*  Step-3L: *M* = -.018 [*d* = -.82]  Step-2L: *M* = -.016 [*d* = -.87]  Step-1L: *M* = -.006 [*d* = -.31]  Step-1R: *M* = -.039 [*d* = -1.55]  Step-2R: *M* = .010 [*d* = .35]  Step-3R: *M* = .069 [*d* = 1.90]  ** EL Lo-ADOS – LL*  Step-3L: *M* = .007 [*d* = .42]  Step-2L: *M* = -.003 [*d* = -.20]  Step-1L: *M* = .003 [*d* = .23]  Step-1R: *M* = .005 [*d* = .25]  Step-2R: *M* = -.014 [*d* = -.65]  Step-3R: *M* = .002 [*d* = .06]  **AlphaNS, mean difference [effect size]**  ** EL Hi-ADOS – EL Lo-ADOS*  Step-3L: *M* = .013 [*d* = .61]  Step-2L: *M* = .001 [*d* = .05]  Step-1L: *M* = -.010 [*d* = -.41]  Step-1R: *M* = -.052 [*d* = -1.61]  Step-2R: *M* = -.005 [*d* = -.19]  Step-3R: *M* = .052 [*d* = 1.26]  ** EL Hi-ADOS – LL*  Step-3L: *M* = -.011 [*d* = -.49]  Step-2L: *M* = -.002 [*d* = -.07]  Step-1L: *M* = -.006 [*d* = -.53]  Step-1R: *M* = -.013 [*d* = -.93]  Step-2R: *M* = -.052 [*d* = -1.72]  Step-3R: *M* = .110 [*d* = 2.49]  ** EL Lo-ADOS – LL*  Step-3L: *M* = -.025 [*d* = -1.39]  Step-2L: *M* = -.003 [*d* = -.16]  Step-1L: *M* = -.003 [*d* = -.18]  Step-1R: *M* = .020 [*d* = .77]  Step-2R: *M* = -.046 [*d* = -1.98]  Step-3R: *M* = .057 [*d* = 1.67]  **AlphaS, mean difference [effect size]**  ** EL Hi-ADOS – EL Lo-ADOS*  Step-3L: *M* = .033 [*d* = 1.57]  Step-2L: *M* = -.001 [*d* = -.07]  Step-1L: *M* = .007 [*d* = .30]  Step-1R: *M* = .003 [*d* = .07]  Step-2R: *M* = -.065 [*d* = -1.99]  Step-3R: *M* = .023 [*d* = .49]  ** EL Hi-ADOS – LL*  Step-3L: *M* = .020 [*d* = .88]  Step-2L: *M* = -.003 [*d* = -.14]  Step-1L: *M* = -.003 [*d* = -.10]  Step-1R: *M* = -.016 [*d* = -.38]  Step-2R: *M* = -.026 [*d* = -.76]  Step-3R: *M* = .027 [*d* = .56]  ** EL Lo-ADOS – LL*  Step-3L: *M* = -.014 [*d* = -.78]  Step-2L: *M* = -.002 [*d* = -.09]  Step-1L: *M* = -.010 [*d* = -.50]  Step-1R: *M* = -.018 [*d* = -.58]  Step-2R: *M* = .039 [*d* = 1.45]  Step-3R: *M* = .005 [*d* = .12] | 59  11^i)^  28^ii)^  20^iii)^ | .445  .050  .449  .050  .264  < .001  .490  .050  .449  .050  .446  .004  .492  .492  .498  .492  .446  .492  .491  .498  .498  .492  .446  .492  .446  .446  .492  .492  .492  .446  .245  .446  .449  .498  .446  .245  .449  .492  .492  .481  .492  .449  .449  .492  .492  .493  .492  .449  .492  .492  .492  .449  .491  .492  .449  .492  .492  .326  .492  .326  .492  .492  .492  .449  .492  .326  .492  .492  .492  .492  .492  .492  .478  .478  .478  .294  .478  .374  .478  .478  .478  .478  .294  .248  .327  .478  .478  .478  .284  .294  .294  .478  .478  .478  .284  .478  .478  .478  .478  .478  .478  .478  .478  .478  .478  .478  .327  .478 |
| Main result | **Results:** *Linking laterality of visual cortical functional connectivity and lateral global motion processing during infancy to later autism* | Table 1 | Main-effect model of  ADOS2 ~ dbWPLI  [“Model 0”]  (OLS regression)  Main-effect model of  ADOS2 ~ dbWPLI + GMLS  [“Model 1”]  (OLS regression)  Comparison of  “Model 0” vs “Model 1”  (LR test) | **Regression coefficient [Effect size]**  *β*(sex_Male) = .59 [part-𝜂^2^ = .043]  *β*(age) = -.02 [part-𝜂^2^ = .084]  *β*(status_LL) = .52 [part-𝜂^2^ = .025]  *β*(F_ThetaNS) = .56 [part-𝜂^2^ = .112]  *β*(F_GammaNS) = -.59 [part-𝜂^2^ = .107]  *β*(F_GammaS) = .39 [part-𝜂^2^ = .055]  *R*^2^ = .322  *β*(sex_Male) = .40 [part-𝜂^2^ = .022]  *β*(age) = -.02 [part-𝜂^2^ = .089]  *β*(status_LL) = .34 [part-𝜂^2^ = .012]  *β*(F_ThetaNS) = .63 [part-𝜂^2^ = .152]  *β*(F_GammaNS) = -.57 [part-𝜂^2^ = .113]  *β*(F_GammaS) = .20 [part-𝜂^2^ = .015]  *β*(GMLS) = .47 [part-𝜂^2^ = .110]  *R*^2^ = .397  **npar \| LogLik \| Δdf \| χ^2^**  Model 0: 6 -73.27  Model 1: 7 -70.77 1 5.014 | 43 | .210  .077  .341  .039  .045  .155  .381  .072  .519  .017  .042  .474  .045  .025 |
| Main result | **Results:** *Linking laterality of visual cortical functional connectivity and lateral global motion processing during infancy to later autism* | Fig. 4a | Bivariate correlation (Pearson’s *r*) | No point removed: *r*(F_GammaS x GMLS) = .30  Influential point removed: *r*(F_GammaS x ADOS2) = .42 | 49  48 | .038  .003 |
| Main result | **Results:** *Linking laterality of visual cortical functional connectivity and lateral global motion processing during infancy to later autism* | Fig. 4a | Main-effect model of  GMLS ~ dbWPLI  (OLS regression) | **Regression coefficient [Effect size]**  *β*(F_GammaS) = .36 [part-𝜂^2^ = .118]  *β*(F_ThetaS) = -.15 [part-𝜂^2^ = .025]  *β*(F_ThetaNS) = -.12 [part-𝜂^2^ = .012]  *β*(N_GammaNS) = .13 [part-𝜂^2^ = .015]  *β*(N_AlphaS) = -.14 [part-𝜂^2^ = .019]  *R*^2^ = .161 | 49 | .021  .301  .475  .423  .365 |
| Main result | **Results:** *Functional connectivity differences between stimulus types and between left and right hemispheres* | Figs. 4b-c  Ext. Data Figs. 3c-d | Across-stimulus difference test of flipped, norm. AoI connectivity  (Permutation test + FDR correct.)  *Notes:*  ^i)^EL-High ADOS  ^ii)^EL-Low ADOS  ^iii)^LL | **Theta, mean difference [effect size]**  ** Social – NonSocial; EL-High ADOS*  Step-3L: *M* = .006 [*d* = .22]  Step-2L: *M* = .006 [*d* = .28]  Step-1L: *M* = .026 [*d* = .99]  Step-1R: *M* = .050 [*d* = 1.31]  Step-2R: *M* = .063 [*d* = 2.09]  Step-3R: *M* = -.151 [*d* = -3.04]  ** Social – NonSocial; EL-Low ADOS*  Step-3L: *M* = -.019 [*d* = -1.37]  Step-2L: *M* = -.047 [*d* = -4.22]  Step-1L: *M* = .010 [*d* = .74]  Step-1R: *M* = -.028 [*d* = -1.49]  Step-2R: *M* = -.010 [*d* = -.67]  Step-3R: *M* = .094 [*d* = 3.83]  ** Social – NonSocial; LL*  Step-3L: *M* = .021 [*d* = 1.17]  Step-2L: *M* = -.033 [*d* = -2.22]  Step-1L: *M* = -.002 [*d* = -.13]  Step-1R: *M* = -.037 [*d* = -1.44]  Step-2R: *M* = .033 [*d* = 1.66]  Step-3R: *M* = .019 [*d* = .57]  **Gamma, mean difference [effect size]**  ** Social – NonSocial; EL-High ADOS*  Step-3L: *M* = .001 [*d* = .06]  Step-2L: *M* = .002 [*d* = .16]  Step-1L: *M* = -.017 [*d* = -1.04]  Step-1R: *M* = -.043 [*d* = -2.40]  Step-2R: *M* = .034 [*d* = 2.03]  Step-3R: *M* = .023 [*d* = .93]  ** Social – NonSocial; EL-Low ADOS*  Step-3L: *M* = .001 [*d* = .11]  Step-2L: *M* = .014 [*d* = 1.98]  Step-1L: *M* = -.010 [*d* = -1.16]  Step-1R: *M* = -.025 [*d* = -2.83]  Step-2R: *M* = .016 [*d* = 1.94]  Step-3R: *M* = .004 [*d* = .34]  ** Social – NonSocial; LL*  Step-3L: *M* = -.009 [*d* = -1.09]  Step-2L: *M* = .007 [*d* = .76]  Step-1L: *M* = -.014 [*d* = -1.31]  Step-1R: *M* = -.004 [*d* = -.37]  Step-2R: *M* = .006 [*d* = .57]  Step-3R: *M* = .015 [*d* = .90]  **Alpha, mean difference [effect size]**  ** Social – NonSocial; EL-High ADOS*  Step-3L: *M* = -.010 [*d* = -.48]  Step-2L: *M* = -.028 [*d* = -1.15]  Step-1L: *M* = .026 [*d* = 1.00]  Step-1R: *M* = .060 [*d* = 1.78]  Step-2R: *M* = -.021 [*d* = -.71]  Step-3R: *M* = -.028 [*d* = -.66]  ** Social – NonSocial; EL-Low ADOS*  Step-3L: *M* = -.030 [*d* = -2.82]  Step-2L: *M* = -.025 [*d* = -2.02]  Step-1L: *M* = .009 [*d* = .70]  Step-1R: *M* = .006 [*d* = .36]  Step-2R: *M* = .038 [*d* = 2.59]  Step-3R: *M* = .002 [*d* = .07]  ** Social – NonSocial; LL*  Step-3L: *M* = -.041 [*d* = -2.90]  Step-2L: *M* = -.026 [*d* = -1.61]  Step-1L: *M* = .016 [*d* = .90]  Step-1R: *M* = .045 [*d* = 1.97]  Step-2R: *M* = -.047 [*d* = -2.37]  Step-3R: *M* = .054 [*d* = 1.88] | 59  11^i)^  28^ii)^  20^iii)^ | .481  .148  .352  .148  .210  .002  .210  .210  .485  .352  .148  .017  .229  .485  .352  .352  .352  .485  .462  .462  .462  .462  .462  .462  .462  .462  .462  .462  .495  .462  .462  .462  .462  .462  .462  .462  .458  .497  .474  .458  .467  .458  .497  .497  .474  .390  .063  .474  .467  .497  .497  .467  .074  .390 |
| Main result | **Results:** *Functional connectivity differences between stimulus types and between left and right hemispheres* | Ext. Data Figs. 3a-b | Across-stimulus difference test of absolute (raw) AoI connectivity  (Permutation test + FDR correct.)  *Notes:*  ^i)^EL-High ADOS  ^ii)^EL-Low ADOS  ^iii)^LL | **Theta, mean difference [effect size]**  ** Social – NonSocial; EL-High ADOS*  Step-3L: *M* = -.008 [*d* = -1.28]  Step-2L: *M* = -.002 [*d* = -.38]  Step-1L: *M* = .004 [*d* = .92]  Step-1R: *M* = .002 [*d* = .39]  Step-2R: *M* = .001 [*d* = .24]  Step-3R: *M* = -.004 [*d* = -.63]  ** Social – NonSocial; EL-Low ADOS*  Step-3L: *M* = .007 [*d* = 1.96]  Step-2L: *M* = .001 [*d* = .46]  Step-1L: *M* = .003 [*d* = 1.26]  Step-1R: *M* = .005 [*d* = 1.61]  Step-2R: *M* = .004 [*d* = 1.22]  Step-3R: *M* = .005 [*d* = 1.41]  ** Social – NonSocial; LL*  Step-3L: *M* = -.002 [*d* = -.47]  Step-2L: *M* = -.004 [*d* = -1.02]  Step-1L: *M* = .000 [*d* = .15]  Step-1R: *M* = -.004 [*d* = -.84]  Step-2R: *M* = -.004 [*d* = -.89]  Step-3R: *M* = .001 [*d* = .20]  **Gamma, mean difference [effect size]**  ** Social – NonSocial; EL-High ADOS*  Step-3L: *M* = .002 [*d* = .36]  Step-2L: *M* = .002 [*d* = .47]  Step-1L: *M* = -.002 [*d* = -.39]  Step-1R: *M* = .000 [*d* = -.13]  Step-2R: *M* = -.001 [*d* = -.33]  Step-3R: *M* = .001 [*d* = .28]  ** Social – NonSocial; EL-Low ADOS*  Step-3L: *M* = .007 [*d* = 2.41]  Step-2L: *M* = .008 [*d* = 2.95]  Step-1L: *M* = .003 [*d* = 1.43]  Step-1R: *M* = .005 [*d* = 2.33]  Step-2R: *M* = .003 [*d* = 1.43]  Step-3R: *M* = .004 [*d* = 2.69]  ** Social – NonSocial; LL*  Step-3L: *M* = .003 [*d* = .88]  Step-2L: *M* = .004 [*d* = 1.04]  Step-1L: *M* = -.001 [*d* = -.23]  Step-1R: *M* = .003 [*d* = 1.15]  Step-2R: *M* = .002 [*d* = .93]  Step-3R: *M* = .002 [*d* = .98]  **Alpha, mean difference [effect size]**  ** Social – NonSocial; EL-High ADOS*  Step-3L: *M* = -.002 [*d* = -.83]  Step-2L: *M* = -.004 [*d* = -1.21]  Step-1L: *M* = .001 [*d* = .25]  Step-1R: *M* = .003 [*d* = .85]  Step-2R: *M* = -.002 [*d* = -.52]  Step-3R: *M* = -.002 [*d* = -.76]  ** Social – NonSocial; EL-Low ADOS*  Step-3L: *M* = -.005 [*d* = -2.86]  Step-2L: *M* = -.004 [*d* = -2.19]  Step-1L: *M* = -.006 [*d* = -2.50]  Step-1R: *M* = -.004 [*d* = -1.56]  Step-2R: *M* = -.001 [*d* = -.35]  Step-3R: *M* = .002 [*d* = 1.35]  ** Social – NonSocial; LL*  Step-3L: *M* = -.003 [*d* = -1.21]  Step-2L: *M* = -.009 [*d* = -3.23]  Step-1L: *M* = -.003 [*d* = -.90]  Step-1R: *M* = -.004 [*d* = -1.21]  Step-2R: *M* = -.009 [*d* = -3.04]  Step-3R: *M* = -.003 [*d* = -1.19] | 59  11^i)^  28^ii)^  20^iii)^ | .  .373  .467  .467  .467  .467  .373  .373  .373  .467  .373  .373  .373  .437  .437  .437  .373  .373  .467  .473  .473  .473  .473  .473  .473  .473  .473  .473  .473  .473  .473  .473  .495  .473  .473  .473  .473  .350  .350  .350  .347  .350  .350  .473  .473  .473  .473  .473  .473  .350  .350  .444  .350  .347  .350 |
| Main result | **Results:** *Functional connectivity differences between stimulus types and between left and right hemispheres* | Fig. 4d | Left-/right-sidedness difference test of dbWPLI variables  (Wilcoxon test + FDR correction)  *Notes:*  ^i)^EL-High ADOS  ^ii)^EL-Low ADOS  ^iii)^LL | **Theta, mean difference [effect size]**  ** Right – Left; NonSocial stimulus*  EL-HiADOS: *M* = -.024 [*d* = -.11]  EL-LoADOS: *M* = .050 [*d* = .47]  LL: *M* = -.021 [*d* = - .23]  ** Right – Left; Social stimulus*  EL-HiADOS: *M* = -.042 [*d* = -.39]  EL-LoADOS: *M* = -.033 [*d* = -.24]  LL: *M* = -.022 [*d* = -.22]  **Gamma, mean difference [effect size]**  ** Right – Left; NonSocial stimulus*  EL-HiADOS: *M* = -.037 [*d* = -.27]  EL-LoADOS: *M* = -.094 [*d* = -1.03]  LL: *M* = -.028 [*d* = -.32]  ** Right – Left; Social stimulus*  EL-HiADOS: *M* = -.053 [*d* = -.38]  EL-LoADOS: *M* = -.067 [*d* = -.82]  LL: *M* = -.074 [*d* = -.82]  **Alpha, mean difference [effect size]**  ** Right – Left; NonSocial stimulus*  EL-HiADOS: *M* = -.079 [*d* = -.68]  EL-LoADOS: *M* = -.053 [*d* = -.50]  LL: *M* = -.018 [*d* = -.23]  ** Right – Left; Social stimulus*  EL-HiADOS: *M* = -.039 [*d* = -.38]  EL-LoADOS: *M* = .047 [*d* = .40]  LL: *M* = -.011 [*d* = -.08] | 59  11^i)^  28^ii)^  20^iii)^ | .623  .131  .623  .616  .616  .677  .897  .004  .616  .616  .031  .131  .419  .616  .616  .616  .616  .989 |
| Supplementary result | **Results:** *Linking laterality of visual cortical functional connectivity and lateral global motion processing during infancy to later autism* | Last paragraph of section | Main-effect model of  ADOS2 ~ GLMS  (OLS regression) | **Regression coefficient [Effect size]**  *β*(sex_Male) = 1.17 [part-𝜂^2^ = .101]  *β*(age) = .00 [part-𝜂^2^ = .000]  *β*(status_LL) = -1.09 [part-𝜂^2^ = .065]  *β*(GMLS) = .53 [part-𝜂^2^ = .074]  *β*(GFLS) = .25 [part-𝜂^2^ = .019]  *β*(LMLS) = .02 [part-𝜂^2^ = .000]  *β*(LFLS) = -.30 [part-𝜂^2^ = .021]  *R*^2^ = .233 | 82 | .005  .898  .026  .018  .236  .914  .210 |

***Abbreviations:*** FC_ = Far-Connectivity; NC_ = Near-Connectivity; [Band]NS = NonSocial stimuli; [Band]S = Social stimuli; dbWPLI = debiased Weighted Phase Lag Index; GMLS = Global Motion Laterality Score; EL = Elevated Likelihood for ASD; LL = Low Likelihood for ASD; LR = Likelihood Ratio; npar = no. of model parameters; LogLik = Log-Likelihood; df = degrees of freedom; GFLS = Global Form Laterality Score; LMLS = Local Motion Laterality Score; LFLS = Local Form Laterality Score


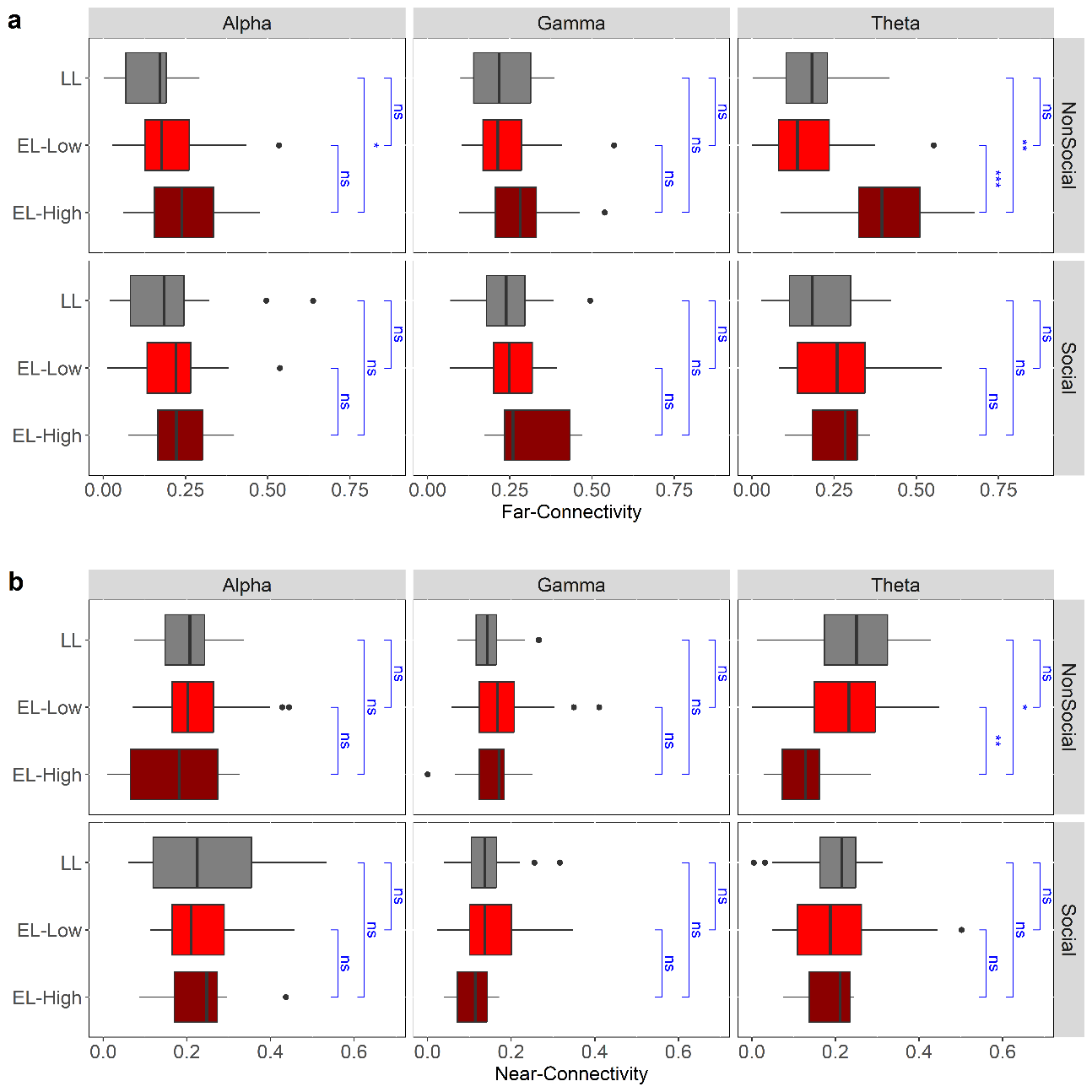


**Supplementary Figure 1 | Group analysis of dbWPLI Near & Far Connectivity variables in six experimental conditions. a.** Group differences in Far Connectivity variables in the six experimental conditions. Significant differences were found in Theta-NonSocial condition between the EL-High ADOS group and the other two groups, but not between these two later groups, and in Alpha Non-Social condition between the two EL groups. No correction was done on the *p*-values here. FDR corrections of *p*-values resulted in the elimination of significance in Alpha-NonSocial but not in Theta-NonSocial conditions (see also **Fig. 3b**). **b.** Group differences in Near Connectivity variables in the six experimental conditions. Significant differences were found in Theta-NonSocial condition between the EL-High ADOS group and the other two groups, but not between these two later groups. Again, no *p*-value correction was done here. FDR corrections of *p*-values resulted in no significant difference in any conditions. See **Supplementary Table 5** for all numerical details. Boxplots show the sample median, and the first and third quartiles; whiskers show minimum and maximum (+ 1.5 s.d.); dots are outliers (+ 1.96 s.d.). (Notes: EL = Elevated Likelihood; LL = Low Likelihood; EL-High = EL-High ADOS; EL-Low = EL-Low ADOS; ns = *p* > .05; * = *p* < .05; ** = *p* < .01; *** = *p* < .001).


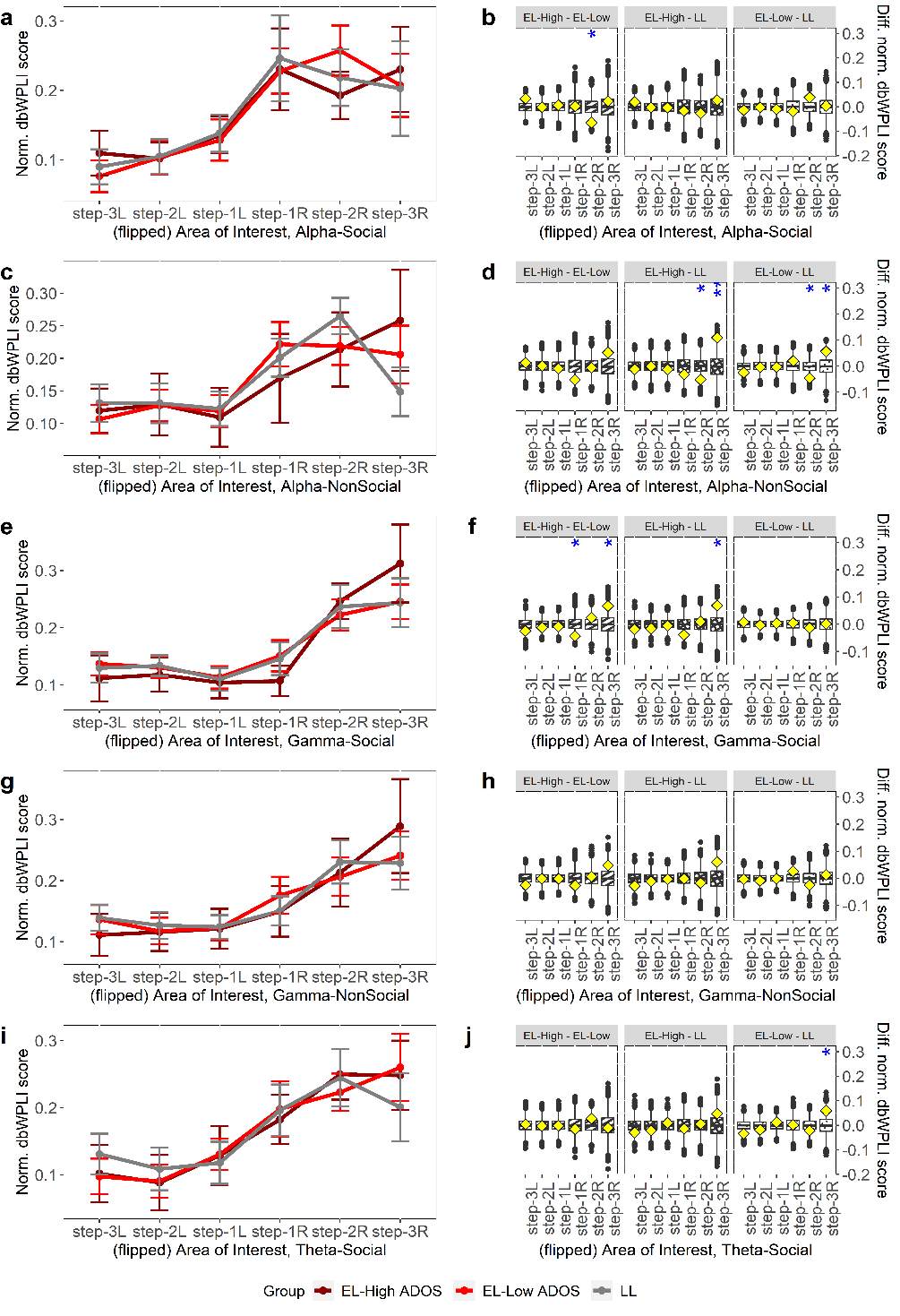


**Supplementary Figure 2 | Permutation tests of across-group differences in five experimental conditions based on the flipped topographies.** Similar to **Figs. 3c**-**d**, but for **a-b.** Alpha-Social condition, showing a difference in step-2R AoI between the EL groups; **c-d.** Alpha-NonSocial condition, showing a difference in step-2R and step-3R AoIs between the each EL group and LL group, but not between the two EL groups; **e-f.** Gamma-Social condition, showing a difference in step-1R and step-3R AoIs between the two EL groups, and in step-3R AoI between the EL-High ADOS and LL groups; **g-h.** Gamma-NonSocial condition, showing no difference; **i-j.** Theta-Social condition, showing a difference in step-3R AoI between the EL-Low ADOS and LL groups. No *p-*value correction was done. FDR correction for multiple testing resulted in no significance found in these 5 conditions (also see **Figs. 3c-d**). Complete numerical details in **Supplementary Table 5**. (*Note*: boxplots are simulated distributions using 10,000 randomization of the group labels, diamonds actual point values in our data, stars mark significance).


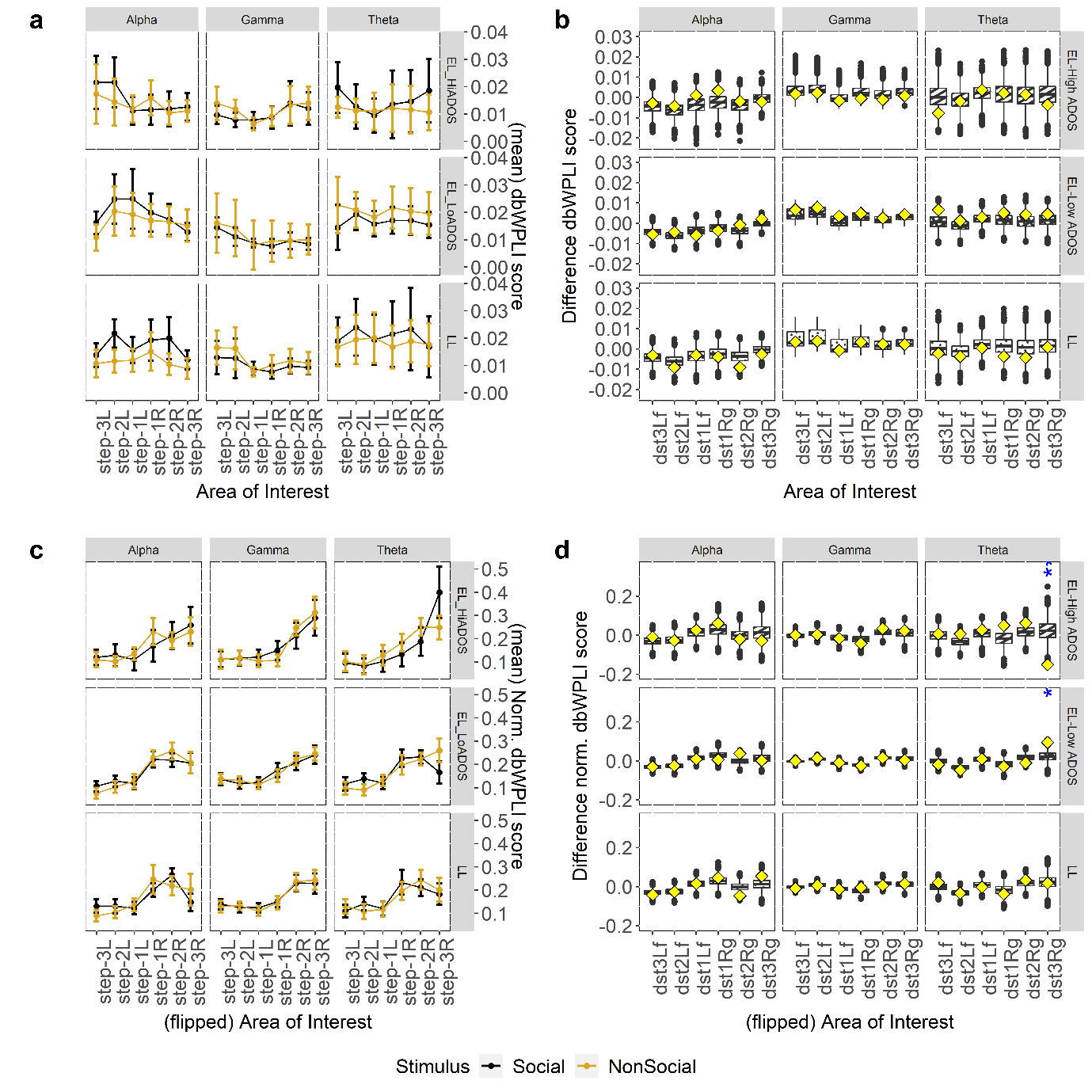


**Supplementary Figure 3 | Across-stimulus topographic connectivity differences in each frequency band and group. a.** Plots of average raw (unnormalized, unflipped) topographic dbWPLI scores across the two stimuli for each group and frequency band. **b.** Permutation tests of across-stimulus differences for the topographies in **a**, showing no significant difference (all *p* > .300) for any group or band (boxplots are simulated distributions using 10,000 randomization of the group labels, diamonds actual point values in our data, stars mark significance). **c-d.** Similar to **a-b**, but for the flipped normalized dbWPLI scores, where a significant difference in 3-step AoI in theta band were revealed for both EL groups (High ADOS: *d* = -3.05, ***p* =.002, *n* = 11; Low ADOS: *d* = 3.82, **p* = .017, *n* = 28) by the permutation tests. All *p*-values were FDR-corrected for multiple testing. (Notes: EL = Elevated Likelihood; LL = Low Likelihood; step-*d*L/-R = step-*d*Left/-Right, *d* = 1, 2, 3; * = *p* < .05; ** = *p* < .01; *** = *p* < .001). Complete numerical details in **Supplementary Table 5**.

**References**

1 Hardiansyah, I. *et al.* Global motion processing in infants’ visual cortex and the emergence of autism. *Communications Biology* **6**, 1-10 (2023).

2 Mullen, E. M. *Mullen scales of early learning*. (AGS Circle Pines, MN, 1995).
